# Supplementary material for: The Effects of Acetazolamide on Cerebral Hemodynamics in Adult Patients with an Acute Brain Injury: A Systematic Review
Source: Brain Sci. 2023 Dec 6;13(12):1678. doi: 10.3390/brainsci13121678 (PMC10741868; doi:10.3390/brainsci13121678)
Supplement: Supplementary file 1 [file brainsci-13-01678-s001.zip › brainsci-2691388-supplementary.pdf]

Supplement S1: Risk of bias of selected studies

Author (year)	Risk of bias (MINORS)	Level of evidence (GRADE)
**Non comparative studies**		
Shinoda et al. (1991)[1]	A clearly stated aim: 2<br>Inclusion of consecutive patients: 1<br>Prospective data collection: 2<br>Endpoints appropriate to the aim of the study: 1<br>Unbiased assessment of the study endpoint: 0<br>Follow-up period appropriate to the aim of the study: 2<br>Loss to follow-up less than 5%: 0<br>Prospective calculation of the study size: 0<br>Total: 8	Low
Kimura et al. (1993)[2]	A clearly stated aim: 2<br>Inclusion of consecutive patients: 1<br>Prospective data collection: 2<br>Endpoints appropriate to the aim of the study: 1<br>Unbiased assessment of the study endpoint: 0<br>Follow-up period appropriate to the aim of the study: 2<br>Loss to follow-up less than 5%: 0<br>Prospective calculation of the study size: 0<br>Total: 8	Low
Yoshida et al. (1996)[3]	A clearly stated aim: 2<br>Inclusion of consecutive patients: 0<br>Prospective data collection: 2<br>Endpoints appropriate to the aim of the study: 2<br>Unbiased assessment of the study endpoint: 0<br>Follow-up period appropriate to the aim of the study: 2<br>Loss to follow-up less than 5%: 0<br>Prospective calculation of the study size: 0<br>Total: 8	Low

Author (year)	Risk of bias (MINORS)	Level of evidence (GRADE)
Szabo et al. (1997)[4]	A clearly stated aim: 2<br>Inclusion of consecutive patients: 0<br>Prospective data collection: 2<br>Endpoints appropriate to the aim of the study: 2<br>Unbiased assessment of the study endpoint: 0<br>Follow-up period appropriate to the aim of the study: 1<br>Loss to follow-up less than 5%: 0<br>Prospective calculation of the study size: 0<br>Total: 7	Low
Jarus-Dziedzic et al. (2011)[5]	A clearly stated aim: 2<br>Inclusion of consecutive patients: 0<br>Prospective data collection: 2<br>Endpoints appropriate to the aim of the study: 2<br>Unbiased assessment of the study endpoint: 0<br>Follow-up period appropriate to the aim of the study: 2<br>Loss to follow-up less than 5%: 0<br>Prospective calculation of the study size: 0<br>Total: 8	Low
**Comparative studies**		
Tanaka et al. (1996)[6]	A clearly stated aim: 0<br>Inclusion of consecutive patients: 0<br>Prospective data collection: 2<br>Endpoints appropriate to the aim of the study: 0<br>Unbiased assessment of the study endpoint: 0<br>Follow-up period appropriate to the aim of the study: 0<br>Loss to follow-up less than 5%: 0<br>Prospective calculation of the study size: 0<br>Adequate control group: 1<br>Contemporary groups: 2<br>Baseline equivalence of groups: 1<br>Adequate statistical analyses: 2<br>Total: 8	Low

Author (year)	Risk of bias (MINORS)	Level of evidence (GRADE)
Kitahara et al. (1996)[7]	A clearly stated aim: 2<br>Inclusion of consecutive patients: 0<br>Prospective data collection: 2<br>Endpoints appropriate to the aim of the study: 1<br>Unbiased assessment of the study endpoint: 0<br>Follow-up period appropriate to the aim of the study: 2<br>Loss to follow-up less than 5%: 0<br>Prospective calculation of the study size: 0<br>Adequate control group: 1<br>Contemporary groups: 2<br>Baseline equivalence of groups: 0<br>Adequate statistical analyses: 1<br>Total: 11	Low
Tanaka et al. (1998)[8]	A clearly stated aim: 2<br>Inclusion of consecutive patients: 1<br>Prospective data collection: 2<br>Endpoints appropriate to the aim of the study: 1<br>Unbiased assessment of the study endpoint: 0<br>Follow-up period appropriate to the aim of the study: 2<br>Loss to follow-up less than 5%: 0<br>Prospective calculation of the study size: 0<br>Adequate control group: 0<br>Contemporary groups: 0<br>Baseline equivalence of groups: 0<br>Adequate statistical analyses: 1<br>Total: 9	Low

Author (year)	Risk of bias (MINORS)	Level of evidence (GRADE)
Chang et al. (2003)[9]	A clearly stated aim: 2<br>Inclusion of consecutive patients: 0<br>Prospective data collection: 2<br>Endpoints appropriate to the aim of the study: 2<br>Unbiased assessment of the study endpoint: 0<br>Follow-up period appropriate to the aim of the study: 2<br>Loss to follow-up less than 5%: 0<br>Prospective calculation of the study size: 0<br>Adequate control group: 1<br>Contemporary groups: 2<br>Baseline equivalence of groups: 2<br>Adequate statistical analyses: 1<br>Total: 14	Low
Nogami et al. (2004)[10]	A clearly stated aim: 2<br>Inclusion of consecutive patients: 0<br>Prospective data collection: 2<br>Endpoints appropriate to the aim of the study: 2<br>Unbiased assessment of the study endpoint: 0<br>Follow-up period appropriate to the aim of the study: 2<br>Loss to follow-up less than 5%: 0<br>Prospective calculation of the study size: 0<br>Adequate control group: 2<br>Contemporary groups: 2<br>Baseline equivalence of groups: 0<br>Adequate statistical analyses: 1<br>Total: 13	Low

Author (year)	Risk of bias (MINORS)	Level of evidence (GRADE)
Bøthun et al. (2019)[11]	A clearly stated aim: 2<br>Inclusion of consecutive patients: 2<br>Prospective data collection: 2<br>Endpoints appropriate to the aim of the study: 1<br>Unbiased assessment of the study endpoint: 2<br>Follow-up period appropriate to the aim of the study: 2<br>Loss to follow-up less than 5%: 2<br>Prospective calculation of the study size: 0<br>Adequate control group: 1<br>Contemporary groups: 2<br>Baseline equivalence of groups: 0<br>Adequate statistical analyses: 2<br>Total: 18	Low

Tab S1

Author (year)	Study design and population	Outcomes	Measuring method	Main results
Shinoda et al. (1991)[1]	Prospective interventional, single center, Japan<br><br>42 patients with SAH	Cerebral perfusion after acetazolamide associated to different findings in the acute and subacute stages of SAH	^{123}I-IMP - SPECT	Diffuse brain swelling: no perfusion in the acute-subacute stages.<br>VMR was reduced in all patients in the acute stage after surgical intervention, but improved over time.<br>VMR decreased more in the subacute stage (9-21 days) and it was more frequently reduced in patient with worse clinical status.
Kimura et al. (1993)[2]	Prospective interventional, single center, Japan<br><br>79 patients with SAH	Correlation between VMR and development of DCI due to vasospasm	^{123}I-IMP - SPECT	Reduced VMR by day 8 after SAH in several territories is associated with DCI due to vasospasm.
Yoshida et al. (1996)[3]	Prospective interventional, single center, Japan<br><br>50 patients with SAH	Relationship between VMR in the acute phase and clinical outcome	Xe-CT	Higher VMR was associated to better clinical outcomes, while there were no differences according to SAH grades or development of vasospasm.
Kitahara et al. (1996)[7]	Prospective interventional, single center, Japan<br><br>22 patients with ICH	VMR in patients with HPH compared to hypertensive patients with no ICH (non-HPH).	Xe-CT	VMR was preoperatively reduced in the ipsilateral hemisphere compared to non-HPH, while in thalamus there were no significant differences. Contralateral VMR in the hemisphere was lower in HPH during in the preoperative phase, compared to non-HPH, to significantly rise chronically.
Tanaka et al. (1996)[6]	Prospective interventional, single center, Japan<br><br>15 patients with ICH	VMR in the chronic stage of putaminal vs thalamic hemorrhages.	Xe-CT	VMR was statistically significant in the chronic stages in both types of hemorrhage.
Szabo et al. (1997)[4]	Prospective interventional, single center, Hungary<br><br>27 patients with aSAH	VMR in a 1-8 years follow-up after vasospasm in aSAH.	TCD	VMR were restored to normal values.

Author (year)	Study design and population	Outcomes	Measuring method	Main results
Tanaka et al. (1998)[8]	Prospective interventional, single center, Japan<br><br>18 patients symptomatic for vasospasm after aSAH and 27 patients asymptomatic for vasospasm after aSAH (tot = 45 patients)	VMR in patients with and without ischemic symptoms due to vasospasm in acute, subacute and chronic stages of aSAH.	Xe-CT	In symptomatic patients, VMR was normal in the acute stage while it was significantly higher in the chronic phase compared to healthy controls (subacute stage was not tested).<br>In asymptomatic patients, VMR was significantly reduced from acute to subacute stage, while it rose chronically.
Chang et al. (2003)[9]	Prospective interventional, single center, Japan<br><br>48 patients with ventriculomegaly after aSAH	VMR in a follow-up (1-12 months) compared to healthy subjects, and after surgical shunting in those patients who developed symptomatic NPH.	Radionuclide angiography with 99mTc-HMPAO	VMR was reduced in patients asymptomatic and symptomatic patients, except in non-responders to surgery. After shunting, VMR was increased in clinically recovered patients while was stable in those who remained symptomatic.
Nogami et al. (2004)[10]	Prospective interventional, single center, Japan<br><br>17 patients with HIE in the subacute stage	Correlation of VMR with clinical outcome;<br>Correlation of MRI findings and clinical outcome;<br>Correlation of VMR with MRI patterns.	Xe-CT	VMR resulted higher in patients with good clinical outcome.<br>Patients with unfavorable MRI pattern (hyperintense lesions in T1 and T2) had lower VMR.
Jarus-Dziedzic et al. (2011)[5]	Prospective interventional, single center, Poland<br><br>24 patients with aSAH	BFV and CVR in a long-term follow-up;<br>CO_2 reactivity in patients treated with clipping, coiling or conservatively.	TCD	VMR after acetazolamide was restored in the chronic stage.<br>Reactivity to CO_2 were preserved in the three groups with not statistically significant differences.
Bøthun et al. (2019) [11]	Prospective interventional, single center, Norway<br><br>42 patients with aSAH and 37 patients with UIA (tot = 79 patients)	CVR as a potential predictor of DCI;<br>Relationship between CVR and rupture status of the aneurysm (UIA vs aSAH).	TCD	Reduction in contralateral VMR is predictive of development of clinical DCI but not radiological infarction.<br>VMR is reduced in patients with aSAH compared to UIA.

^{123}I-IMP - SPECT: N-isopropyl-^{123}iodine Single Photon Emission Computed Tomography; 99mTc-HMPAO: 99mTc-hexamethylpropyleneamineoxime; aSAH: aneurysmatic Subarachnoid Hemorrhage; DCI: Delayed Cerebral Ischemia; DFV: Diastolic Flow Velocity; HIE: Hypoxic-Ischemic Encephalopathy; HPH: hypertensive putaminal hemorrhage; ICH: Intracranial Hemorrhage; MFV: Mean Flow Velocity; MRI: Magnetic Resonance Imaging; PI: Pulsatility Index; RI: Resistivity Index; SAH: Subarachnoid Hemorrhage; SFV: Systolic Flow Velocity; TCD: transcranial doppler; VMR: cerebral vasomotor reactivity; Xe-CT: Xenon Computed Tomography; UIA: Unruptured Intracranial Aneurysm

References

[1] J. Shinoda, T. Kimura, T. Funakoshi, Y. Araki, and Y. Imao, “Acetazolamide Reactivity on Cerebral Blood Flow in Patients with Subarachnoid Haemorrhage,” 1991.

[2] Kimura T, Shinoda J, and Funakoshi T, “Prediction of cerebral infarction due to vasospasm following aneurysmal subarachnoid haemorrhage using acetazolamide-activated 123I-IMP SPECT,” *Acta Neurochir (Wien)*, vol. 123, pp. 125–128, 1993.

[3] K. Yoshida, S. Nakamura, H. Watanabe, and K. Kinoshita, “Early cerebral blood flow and vascular reactivity to acetazolamide in predicting the outcome after ruptured cerebral aneurysm,” *Acta Neurol Scand*, vol. 93, no. SUPPL166, pp. 131–134, 1996, doi: 10.1111/j.1600-0404.1996.tb00576.x.

[4] Szabo S, Sheth RN, Novak L, Rozsa L, and Ficzere A, “Cerebrovascular reserve capacity many years after vasospasm due to aneurysmal subarachnoid hemorrhage. A transcranial Doppler study with acetazolamide test.,” *Stroke*, vol. 28, no. 12, pp. 2479–2482, Dec. 1997.

[5] K. Jarus-Dziedzic, M. Głowacki, A. Warzecha, J. Jurkiewicz, Z. Czernicki, and E. Fersten, “Cerebrovascular reactivity evaluated by transcranial doppler sonography in patients after aneurysmal subarachnoid haemorrhage treated with microsurgical clipping or endovascular coiling technique,” *Neurol Res*, vol. 33, no. 1, pp. 18–23, Jan. 2011, doi: 10.1179/016164110X12700393823534.

[6] Tanaka A, Yoshinaga S, Nakayama Y, Kimura M, and Tomonaga M, “Cerebral blood flow and clinical outcome in patients with thalamic hemorrhages: a comparison with putaminal hemorrhages,” *J Neurol Sci*, vol. 144, pp. 191–197, 1996.

[7] T. Kitahara, T. Yamashita, S. Kashiwagi, N. Kawakami, H. Ishihara, and H. Ito, “Hemodynamics of hypertensive putaminal hemorrhage evaluated by xenon-enhanced computed tomography and acetazolamide test,” *Acta Neurol Scand*, vol. 93, no. SUPPL166, pp. 139–143, 1996, doi: 10.1111/j.1600-0404.1996.tb00579.x.

[8] A. Tanaka, S. Yoshinaga, Y. Nakayama, and M. Tomonaga, “Cerebral Blood Flow and the Response to Acetazolamide during the Acute, Subacute, and Chronic Stages of Aneurysmal Subarachnoid Hemorrhage,” *Neurologia medico-chirurgica (Tokyo)*, vol. 38, pp. 623–632, 1998.

[9] C. C. Chang, N. Kuwana, S. Ito, T. Yokoyama, H. Kanno, and I. Yamamoto, “Cerebral haemodynamics in patients with hydrocephalus after subarachnoid haemorrhage due to ruptured aneurysm,” *Eur J Nucl Med Mol Imaging*, vol. 30, no. 1, pp. 123–126, Jan. 2003, doi: 10.1007/s00259-002-1032-x.

[10] K. Nogami *et al.*, “Analysis of magnetic resonance imaging (MRI) morphometry and cerebral blood flow in patients with hypoxic-ischemic encephalopathy,” *Journal of Clinical Neuroscience*, vol. 11, no. 4, pp. 376–380, 2004, doi: 10.1016/j.jocn.2002.12.006.

[11] M. L. Bøthun *et al.*, “Impaired cerebrovascular reactivity may predict delayed cerebral ischemia after aneurysmal subarachnoid hemorrhage,” *J Neurol Sci*, vol. 407, Dec. 2019, doi: 10.1016/j.jns.2019.116539.
